# The implementation of the serial trial intervention for pain and challenging behaviour in advanced dementia patients (STA OP!): a clustered randomized controlled trial

**DOI:** 10.1186/1471-2318-11-12

**Published:** 2011-03-24

**Authors:** Marjoleine JC Pieper, Wilco P Achterberg, Anneke L Francke, Jenny T van der Steen, Erik JA Scherder, Christine R Kovach

**Affiliations:** 1 EMGO+ Institute for Health and Care Research, van der Boechorststraat 7, 1081 BT Amsterdam, The Netherlands; 2 Department of Nursing Home Medicine, VU University Medical Center Amsterdam, van der Boechorststraat 7, 1081 BT Amsterdam, The Netherlands; 3 Department of Public Health and Primary Care, Leiden University Medical Center, Postzone V0-P, PO Box 9600, 2300 RC Leiden, The Netherlands; 4 Netherlands Institute for Health Services Research (NIVEL), PO Box 1568, 3500 BN Utrecht, The Netherlands; 5 Department of Clinical Neuropsychology, VU University Amsterdam, van der Boechorststraat 1, 1081 BT Amsterdam, The Netherlands; 6 University of Wisconsin-Milwaukee, Milwaukee, Wisconsin, USA

## Abstract

**Background:**

Pain (physical discomfort) and challenging behaviour are highly prevalent in nursing home residents with dementia: at any given time 45-80% of nursing home residents are in pain and up to 80% have challenging behaviour. In the USA Christine Kovach developed the serial trial intervention (STI) and established that this protocol leads to less discomfort and fewer behavioural symptoms in moderate to severe dementia patients. The present study will provide insight into the effects of implementation of the Dutch version of the STI-protocol (STA OP!) in comparison with a control intervention, not only on behavioural symptoms, but also on pain, depression, and quality of life. This article outlines the study protocol.

**Methods/Design:**

The study is a cluster randomized controlled trial in 168 older people (aged >65 years) with mild or moderate dementia living in nursing homes. The clusters, Dutch nursing homes, are randomly assigned to either the intervention condition (training and implementation of the STA OP!-protocol) or the control condition (general training focusing on challenging behaviour and pain, but without the step-wise approach). Measurements take place at baseline, after 3 months (end of the STA OP! training period) and after 6 months.

Primary outcome measures are symptoms of challenging behaviour (measured with the Cohen-Mansfield Agitation Inventory (CMAI) and the Neuropsychiatric Inventory-Nursing Home version (NPI-NH)), and pain (measure with the Dutch version of the Pain Assessment Checklist for Seniors (PACSLAC-D) and the Minimum Data Set of the Resident Assessment Instrument (MDS-RAI) pain scale). Secondary outcome measures include symptoms of depression (Cornell and MDS-RAI depression scale), Quality of Live (Qualidem), changes in prescriptions of analgesics and psychotropic drugs, and the use of non-pharmacological comfort interventions (e.g. snoezelen, reminiscence therapy).

**Discussion:**

The transfer from the American design to the Dutch design involved several changes due to the different organisation of healthcare systems. Specific strengths and limitations of the study are discussed.

**Trial registration:**

Netherlands Trial Register (NTR): NTR1967

## Background

The number of dementia patients in the Netherlands is estimated to be 235,000. Due to the aging population and the rising life expectancy, this number may exceed 500,000 in 2050. In 2000, around 30% of the people with dementia were living in nursing homes [1,2]. In nursing home residents, pain is highly prevalent: at any given time, 45-80% of the residents are in pain [3-6]. The most prominent features of pain in dementia patients are challenging behaviour, such as vocalizations (crying, screaming), noisy breathing, facial expressions (e.g. grimacing), restless or strained body expressions, aggressiveness and resistance to care [7,8]. These patterns of behaviour (like delusions, hallucinations, agitation/aggression, dysphoria/depression, anxiety, euphoria/elation, apathy/indifference, disinhibition, irritability/lability, aberrant motor behaviour, night-time disturbances, and appetite/eating disturbances) are also known as neuropsychiatric symptoms of dementia, which are a prominent feature of dementia: in nursing homes up to 80-85% of the residents have one or more of these (clinically relevant) neuropsychiatric symptoms, confusingly often also referred to as challenging behaviour [9-11].

Studies consistently show that patients with dementia are undertreated for pain [3,12-15]. Research indicates that both pharmacological interventions (analgesic medication) and non-pharmacological comfort measures are underutilized [16-18]. There are many reasons for the undertreatment of pain in this population. First, there are difficulties in the assessment of pain and challenging behaviour; the verbal communication of patients with severe dementia is often limited or completely lacking, and behavioural symptoms may provide the only indications for pain, affective discomfort or unmet needs. Second, agitated behaviour in dementia patients may point to pain [19], but it can also be related to affective discomfort. This agitated behaviour is often treated with psychotropic drugs (antipsychotic or anxiolytic medication) with several adverse effects, like drowsiness, depressed mood and falls [20].

A clinical protocol, the STI - Serial Trial Intervention (first referred to as: Assessment and treatment of Discomfort in Dementia or ADD-protocol) - was developed in the United States to address the problems in assessment and management of pain and challenging behaviour in people with dementia [8,21-23]. It is based on the 'unmet need' theoretical framework, in which behaviour can be seen as a way for cognitively impaired people to express their unmet physical and affective needs [24,25]. It is a stepped care protocol, i.e. if in one step the assessment is negative, or if targeted interventions fail to decrease symptoms, one moves to the next step. The protocol is specifically designed for dementia patients with moderate to severe cognitive impairment, because in this particular group verbal communication is often impaired and healthcare professionals therefore have to rely (at least partly) on behavioural symptoms. Since it is often unclear whether these behavioural symptoms are a result of pain or affective discomfort, a systematic approach for exploring and managing the symptoms is needed.

### The STI-protocol consists of five steps

**Figure 1:**
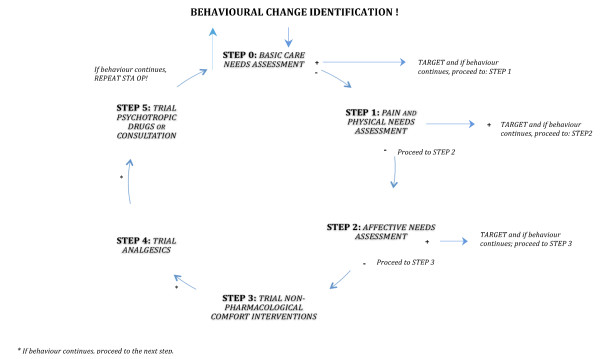
**the STI and STA OP! intervention**.

The FIRST step is to perform a physical needs assessment that focuses on probable causes of behavioural symptoms related to pain or affective discomfort. The SECOND step is to perform a needs assessment that focuses on affective needs of people with dementia. The THIRD step concerns a trial of non-pharmacological comfort interventions, and the FOURTH step a trial of analgesics. STEP FIVE refers to consultation of other health care professionals or practitioners, or a trial of psychotropic drugs (see Figure 1).

The effects of the protocol on patient outcomes and professionals' interventions have been investigated in the USA [8,21,23]. The most recent and well-designed study [23] used a double-blinded randomized design and tested the effects of the protocol in subjects with moderate to severe dementia. The researchers established a significant decrease in the number of discomfort symptoms, measured with the Discomfort Scale - Dementia of Alzheimer type (DS-DAT) [26]. The study however did not assess pain with a measure developed for pain assessment in dementia (an observational pain instrument), quality of life, and depression. Another limitation of that study was that there was insufficient blinding of the outcome measurement.

Furthermore, it is unclear if the protocol can be effectively used in other countries, with a different other long term care (LTC) organisation. The organisation, availability and level of education of staff and availability of additional resources differ across settings and countries [27]. Dutch nursing homes, for instance, are differently organized than those in the USA [27,28]: a typical Dutch nursing home accommodates 150-200 residents, has specialized psychogeriatric wards for dementia patients, the nurses have had a longer training than in the USA, trained nursing home physicians (employed by the nursing home) provide medical care, and most nursing homes have also psychologists in their staff.

Therefore, in order to evaluate the STI in this setting, some changes in the original protocol were executed. In the original protocol for example, nurses performing the physical examination, while in Dutch nursing homes this is a task of a nursing home physician. For the analysis and care planning of behavioural problems, we introduced the expertise of the psychologist. The translated and revised version of the STI has been named STA OP!

#### Research aims and hypotheses

The purpose of this study is to assess the effects of the stepped wise Dutch version of the STI (STA OP!) versus a non-stepped wise approach to pain and behavioural symptoms.

Research questions:

1. Does STA OP! affect the number of symptoms of pain or challenging behaviours in nursing home residents differently (compared to usual care)?

2. Does the use of STA OP! lead to a change in use of analgesics and psychotropic drugs?

3. Does the use of STA OP! result in a change in use of non-pharmacological comfort interventions?

4. Does the use of STA OP! lead to a change in depressive symptoms and the quality of life in advanced dementia patients?

5. Is the effect of intervention moderated by the Apolipoprotein Epsilon 4 (Apo-E4) status of the patient?

It is hypothesized that implementation of the STA OP!-protocol will lead to less pain, fewer expressions of challenging behaviour, improved mood, higher use of pain medication and a lower use of antipsychotics (resulting in a decrease in side effects) with an increased use of non-pharmacological comfort interventions. However, we will also be able to identify findings of outcomes that reverse the direction of the hypothesis.

## Methods

### Study design

The study is a cluster randomized controlled trial. The clusters, Dutch nursing homes, are randomly assigned to either the intervention condition (implementation of the STA OP!-protocol) or the control condition (general training without the step-wise approach). Measurements take place at baseline, after 3 months (at the end of the STA OP!-training period) and after 6 months.

The study is single blinded i.e. the researcher will know the condition, while the research assistants performing the measurements and the persons analysing and score the video registrations are blinded. They will also not be informed about the specific research questions and conditions.

### Procedure

#### Recruitment

Institutions and nursing homes will be recruited within the 'University Network for Organisations of Elderly care' of the VU University Medical Center (UNO-VUmc). The aim of this collaboration network is to build greater knowledge about the best multidisciplinary care for vulnerable elderly. The network provides an infrastructure in which patient care/health care innovations, education and research can take place. Cooperation between institutions and between the institutions and the university will lead to more knowledge and better care. More than half of the eighteen member institutions will participate in this study.

#### Inclusion criteria of units

The nursing homes participating in this study will be randomized in an intervention or a control condition. Nursing homes participating in this study have to meet the following criteria: at least one psychogeriatric unit willing to participate and no major organizational changes or building activities are planned or performed in the study period. Facilities are invited to participate in the study, and after approval/inclusion randomized into control or intervention.

#### Randomization

A separate number will be assigned in order of registration to every institution, nursing home and/or participating unit. When an institution is participating with multiple affiliated nursing homes and/or units, we will investigate whether there are sources of possible contamination by professionals. Contamination is possible, if a professional (e.g. nurse, physician or psychologist) is operating in various nursing homes and/or units within the institution. If so, these different affiliated nursing homes and/or units will be randomized as one. In the event that an institution with multiple affiliated nursing homes and/or unit is participating in the study and there is no contamination by professionals, these locations and/or units will be randomized as separate participants. The randomization will take place by an independent researcher using the program "Random Allocation Software", made available by the EMGO+ Institute for Health and Care Research in Amsterdam.

#### Informed consent, inclusion and exclusion criteria

Informed proxy consent will be obtained for all residents by family/caregivers. Residents will then be screened on dementia severity, behavioural problems and indications of pain.

#### Inclusion and exclusion criteria (residents) (Table 1)

**Table 1:** Instruments and the different assessment points

	Instruments	When & who?	Baseline	3 months (= ca. 12-13 weeks)	6 months (= ca. 26 weeks)
*Selection criteria*	- GDS (Reisberg)	Pre selection	NHP		
	- MDS-CPS	Pre selection	NHP		
					
*Primary Outcome*	- CMAI	Incl.	Nurse	Nurse	Nurse
	- MDS-RAI pain scale	Incl.	Nurse	Nurse	Nurse
	- NPI-NH	Incl.	Nurse	Nurse	Nurse
	- PACSLAC-D	Incl.	Nurse & VIDEO	Nurse & VIDEO	Nurse
					
*Secondary Outcome*	- Qualidem	Incl.	Nurse	Nurse	Nurse
	- Cornell & MDS-DRS	Incl.	Nurse	Nurse	Nurse
	- Medication	Incl.	RA	RA	RA
	- DS-DAT	Incl.	VIDEO	VIDEO	
	- FACS	Incl.	VIDEO	VIDEO	
					
*Control variables/Demographics*	- (co-) Morbidity	Incl.	NHP		
	- Hospitalisation, fixation etc.	Incl.	RA	RA	RA
	- Demographics; sex, age, background	Incl.	RA		
	- ADL (Katz)	Incl.	Nurse		
	- Apolipoprotein E4	Incl.	RA		

NHP = Nursing Home Physician, Nurse = caregiver, RA = research assistant, Incl. = included residents

Residents to be entered into the study meet the following (ABC) pre-selection criteria:

A) Moderate to severe cognitive impairment, according to the Global Detoriation Scale (GDS) [29]. Those residents with a GDS score of 5, 6 or 7 will be eligible for this study.

B) No chronic psychiatric diagnosis other than a dementia associated diagnosis.

The GDS score and also the absence or presence of a chronic psychiatric score (see criteria 1 and 2), will be assessed by the nursing home physician.

Proxy consent will be obtained for all residents meeting these two criteria (A & B). However, residents who are to be actively enrolled in the experimental or control condition will have to meet an additional selection criterion.

C) The criterion to enter the study protocol is the amount of clinically significant symptoms of pain and/or challenging behaviour. Inclusion criteria are: a score of at least 44 on the Dutch version of the Cohen-Mansfield Agitation Inventory (CMAI) [30,31]or a score of at least 4 (frequency × severity) on items of the Neuropsychiatric Inventory - Nursing Home Version (NPI-NH) [32,33]or an indication of clinically relevant pain (intensity × frequency ≥ 2) according to the Minimum Data Set of the Resident Assessment Instrument (MDS-RAI)-pain scale [34] in measurement week 0 (baseline).

The following data will be gathered for all the included residents: Pain observation (Dutch version of the Pain Assessment Checklist for Seniors; PACSLAC-D) [35-37] Depression (Cornell and MDS-RAI depression scale) [38,39] Quality of life (Qualidem) [40-42], ADL (Katz) [43], co-morbidity, demographics and Apolipoprotein Epsilon 4 (from Buccal mucosa swabs). In addition, 5-minutes video recordings will be made for a blinded scoring of pain and/or discomfort.

This inclusion procedure will take place on units in the intervention condition, as well on units in the control condition. These control units will be instructed to treat the included residents with 'standard care'.

#### 'Clinical' inclusion of residents

Those residents who initially do not score above the threshold on the NPI-NH, MDS-RAI pain scale or the CMAI, but who have a change in behaviour after the instrument screening, can be included in the study (after contacting the researcher and after obtaining proxy informed consent). Newly admitted residents on the unit can, after a six week 'adjustment period', follow the same procedure. The resident will (again) be assessed with the same instruments as the regular residents included, after approval by the researcher. Final analysis will be performed with and without these additional groups.

### Sample size and power analysis

One of the primary outcome measures in this study is the CMAI, a behavioural observation scale with 29 behavioural items. Each item may be scored between 1 and 7, depending on the frequency of existing symptoms. The range on the CMAI is 29-203. In Dutch nursing homes the median is 44 [44]; there is no normal distribution, but the distribution of the change is more or less normally distributed. The standard deviation of difference scores (difference of 4) is 13. The expected difference is however larger (at least 10 points). To detect a 15% difference between the intervention (STA OP!-protocol) and control condition with an α = .05 and β = .80, 56 residents will be required. However, in cluster randomization, a design effect should be taken into account; clustering of items (consistency of CMAI items [ICC] among residents in a unit is .10) and also the effect of large differences in sample sizes per cluster enhances the design effect [45].

In this study the design effect was estimated at 1.5; So, 1.5 × 56 = 84 residents (after 4 weeks) will be needed in total to detect a 15% difference with an α = .05 and β = .80. However, we expect a 50% dropout due to non-response and loss to follow-up, i.e. we will need 168 patients in total; 84 in the intervention- and 84 in the control group.

### Intervention condition: implementation of STA OP!

A comprehensive training program for healthcare professionals will be organized to implement the STA OP!-protocol. Healthcare professionals participating in this study will be nursing home physicians, psychologists, occupational therapists and registered or certified nurses (CNA/RN). In most of the nursing homes the majority of the registered or certified nurses will have relatively low educational levels 1, 2 or 3, which in Dutch is called 'verzorgenden' or 'helpenden'. Nurses and physicians who only work night shifts will be excluded from this study. Only the CNA/RN (levels 3, 4 or 5) is authorized to start the protocol.

Nursing home staff from all experimental units will be trained in the stepwise working method of the STA OP!-protocol in five meetings lasting three hours each. The team members will be taught enhanced physical and affective assessment skills that target needs commonly found in people with advanced dementia. A collaborating training centre, with very experienced trainers, will provide the STA OP!-training. These trainers are advanced practice nurses or have other medical backgrounds and have specific professional expertise regarding dementia, pain and discomfort.

In the sessions it will be discussed how nursing home staffs can recognize symptoms related to pain and affective discomfort, and how they can communicate with each other about these symptoms. In the first meetings the initial two steps of the STA OP!-protocol will be discussed, while in the follow up meetings the focus is on the last three steps.

The steps of the STA OP!-protocol are outlined in the following section. Each step is systematically written down in the STA OP!-protocol, which serves as a template for nurses and nursing home physicians to use the stepwise approach in practice (see also Figure 1). How much time it takes to decide to proceed to the subsequent step, depends on the specific interventions chosen. However, in general when effects are lacking or when they are limited, this should not take longer than one week.

Other healthcare professionals can be consulted at any time and at each step in the process.

#### • Step 0: Basic care needs

The nurse assesses whether or not basic physical care needs are fulfilled. Basic care needs concern, for instance hunger, thirst, a need for glasses, hearing aids or toileting.

#### • Step 1: Physical needs assessment (nurse and nursing home physician)

In addition to a brief physical nursing assessment, the nurse will also fill out an observational pain instrument (PACSLAC-D). If these assessments are negative, a nursing home physician (or if available a nurse practitioner) performs a more comprehensive physical assessment to find other probable physical causes, such as inflammation, infection, acute illness or a chronic condition possibly responsible for the observed behaviour.

For those residents already using pain medication or psychotropic drugs and still have behavioural symptoms possibly related to pain or affective discomfort, the nursing home physician will also assess whether the medication given is in accordance with the guidelines of the World Health Organization (WHO) and Verenso (the Dutch association of nursing home physicians) (also see Step 4 and 5).

#### • Step 2: Affective needs assessment

By using a needs-oriented and tailored approach, the nurse assesses possible problems regarding environmental stress, a possible imbalance between sensory stimulating and sensory calming activities, or a lack in meaningful human interactions. The psychologist working in the nursing home can be consulted at this step.

#### • Step 3: Non-pharmacological comfort interventions

In this step non-pharmacological comfort interventions will be conducted and implemented, in line with the personal history of the resident. Examples of comfort interventions are soothing, supportive verbal communication, supportive touch, and sensory stimulation by music, nice smells or soft materials. A study by van Weert [46] showed that these types of comfort interventions are effective in reducing discomfort in nursing home residents.

#### • Step 4: Trial of analgesics

In this step of the protocol the nursing home physician is advised to prescribe a trial of analgesics according to the validated pain ladder, developed by the World Health Organization [47]. Specific guidelines for use in the elderly are given to each participating nursing home physician in a training session, and similarly for physicians working in control and intervention units.

#### • Step 5: Consultation of relevant other disciplines (e.g. psychiatrist) and/or trial of prescribed psychotropic drugs

In the protocol and training sessions, nursing home physicians are instructed to use the guidelines of the Dutch association of nursing home physicians (Verenso) for prescribing psychotropic medication [48]. The Verenso-guidelines clearly describe how and when psychotropic drugs are beneficial for dementia patients. In general, it is believed that this medication is only indicated for specific symptoms and for a fixed period of time.

In the training session it is also discussed that in general, a subsequent protocol step is needed when targeted assessments are negative, or when the symptoms related to pain or affective discomfort have not been reduced sufficiently by the targeted interventions.

During the training, the nurses and nursing home physicians start using the STA OP!-protocol for the residents who have been assessed by the blinded research assistants at pre-test (in week 0) as having a CMAI, NPI-NH and/or MDS-RAI pain scale score above the threshold. It can be expected that the STA OP!-protocol will also be used in other residents showing behavioural symptoms of pain or affective discomfort (outside the measurement weeks): this is allowed and will be registered. To promote the use of the protocol in practice, a focus group will be formed within the units of the institution. The researcher performs site-visits ones a week on the experimental units and provides nurses and nursing home physicians feedback on their use of the STA OP!-protocol in particular residents and answers their questions regarding pain or affective discomfort.

### Control condition

In the control condition there is also a training program for healthcare professionals/nurses focusing on challenging behaviour and pain, but *without *the stepwise approach.

The nursing home physicians (and nurse practitioners if available) in the experimental and control group receive, besides the training sessions focusing on challenging behaviour and pain (with or without a stepwise approach), both exactly the same training regarding the (evidence based) treatment of pain and challenging behaviour. The training in geriatric pain management focuses on appropriate short- and long-acting drugs for the treatment of acute and chronic pain, dose escalation, analgesic escalation and management of side effects. The training in geriatric behaviour treatment focuses on the appropriateness of the use of psychotropic medication for several indications and its side effects. In this additional training it is stressed that other interventions are often more appropriate. This training is given by an experienced nursing home physician (WA).

Next to these two training programs, there is one important change in the control condition from the standard care situation: the nurses and nursing home physicians are informed which residents of their units have a CMAI, NPI-NH or MDS-RAI pain scale score higher than threshold at pre-test (week 0), according to the assessments of the nurses and blinded research assistants.

### Therapy compliance

In the proposed study, therapy compliance is taken into account by the fact that the research assistant/advanced practice nurse who visits the experimental units once a week will have ample and structured focus on the actual use of the protocol in practice. The site-visits will include assessment whether the protocol is adequately used, the patient assessments performed adequately, if planned non-pharmacological interventions are executed and if prescribed analgesics or psychotropic drugs are indeed administered and used. The daily logs from the units will provide additional information on the actual use of the protocol, as well as reports from interdisciplinary meetings.

### Instruments

Various baseline measurements, primary outcome measures, secondary outcome measures, demographic-, and control variables can be distinguished in this study. The instruments are observational instruments, which are filled in by a contact nurse. In addition, video recordings will be made for analysis by independent blinded observers on pain and discomfort (see Table 1).

### Primary outcomes

Primary outcomes measures include symptoms of challenging behaviour and/or pain.

#### Challenging behaviour

Symptoms of challenging behaviour will be measured with the Dutch version of the

Cohen-Mansfield Agitation Inventory (CMAI) [30,31,49,50], and with the Dutch version of the Neuropsychiatric Inventory-Nursing Home Version (NPI-NH) [51-53].

The CMAI was developed to assess agitation in nursing home patients [30,31]. It is more specific than general-purpose behaviour rating scales, which usually include self-care activities, cognition, and mood items. The CMAI uses a 7-point scale to assess the frequency (ranging from 'never' to 'several times an hour') of 29 behaviours commonly seen in nursing home residents. Behaviours are characterized in four clusters: verbally aggressive (e.g., directed at a person or object), verbally nonaggressive (not directed at a specific object or person), physically aggressive (directed), and physically nonaggressive (undirected). The total score is most commonly used to quantify behavioural disturbance. Professional caregivers, who are usually nurses or nursing assistants, assess this scale. The staffs are trained prior to using the instrument. Factor analysis has shown three basic dimensions underlying the 29 CMAI items: physical aggression, physical nonaggression, and verbal agitation. It is a reliable and valid instrument to measure behavioural symptoms in dementia patients [49,50].

The NPI-NH is a structured interview with a healthcare professional (CNA/RN). In this interview ten neuropsychiatric symptoms are assessed: delusions, hallucinations, agitation/aggression, dysphoria, anxiety, euphoria, apathy, disinhibition, irritability/lability. Screening questions are asked to determine whether behavioural changes are present. In the case of a positive answer, further questions are asked and the severity and frequency of the behavioural disturbances are determined. The Dutch version of the NPI has high inter-rater agreement and is found to be a valid rating scale for measuring a wide range of behavioural and psychological symptoms of dementia [51,52].

#### Pain

Symptoms of pain will be measured with the pain scale of the Dutch version of the Minimum Data Set of the Resident Assessment Instrument (MDS-RAI) [34] and with the Pain Assessment Checklist for Seniors (PACSLAC-D) [37]. The MDS contains two pain items: 'pain frequency' and 'pain intensity'. In the MDS, pain frequency is coded as no pain (0); less than daily pain (1); and daily pain (2) in the last 7 days. Pain intensity is categorized as no pain; mild pain (0); moderate pain (1); and severe pain (2, times when pain is horrible or excruciating) in the last 7 days. The product of intensity and severity in this study has to be ≥2 to be defined as clinically relevant pain.

The validity and precision of pain measurement with MDS items have been established against the Visual Analogue Scale in a study involving 95 US nursing home residents [34]. The definition of pain in the MDS is ''Pain refers to any type of physical pain or discomfort of the body. Pain may be localized to one area, or be more generalized. It may be acute or chronic, continuous or intermittent (comes and goes), or occur at rest with movement. The pain experience is very subjective; pain is whatever the resident says it is." Coding instructions are ''Code for the highest level of pain present in the last seven days" [54].

The PACSLAC-D is a brief and manageable version of the Pain Assessment Checklist for Seniors with Limited Ability to Communicate [35] in Dutch that was developed by Zwakhalen [37], with a three component solution including 24 items. This version had high levels of internal consistency for the complete scale (Cronbach's alpha range 0.82-0.86) and for all subscales (alpha range 0.72-0.82).

Both the pain scale of the MDS-RAI and the PACSLAC-D will be assessed by the contact nurse of a particular resident at baseline and after 3 and 6 months.

#### Video registrations

At baseline, 5 minute standardized video recordings of the included patients will be made during both morning care and mealtime. This will be repeated after three months.

Independent, blinded raters will score the video-recordings, by using the PACSLAC-D. These video-recordings will also be used to independently code another pain and discomfort scale, the Dutch version of the Discomfort Scale- dementia of Alzheimer Type (DS-DAT) [26,55]. The DS-DAT is an observational scale with 9 items, and measures symptoms regarding vocalizations, breathing, facial expression, and body movement. The reliability and validity of the Dutch version has been established as high [55-57]. In addition, the video-recordings will be analyzed using the Facial Action Coding System (FACS) [58].

### Secondary outcomes

Secondary outcomes include symptoms of depression, an indication of the quality of life and changes in prescriptions of medication.

#### Depressive symptoms

Depressive symptoms will be measured with the Cornell depression scale and the depression rating scale of the Minimum Data Set Depression Rating Scale (MDS-DRS).

The MDS-DRS is a seven-item scale, with all items scored as, 0 (indicator not exhibited), 1 (indicator of this type exhibited at least once in the last 30 days and up to 5 days a week) or 2 (indicator exhibited daily or almost daily) [59]. The scores range between 0 and 14. The mood-items in the MDS 2.0 have good inter-rater reliability [54,60,61]. (With a cut-off point of 3, it differentiates well between residents with few or many depressive symptoms. Compared to (DSM-IV) psychiatric criteria for depression it has a high sensitivity (91%), and a lower specificity (69%) [59].

The Cornell Scale is a well-known, mostly caregiver-rated scale that is particularly suited to differentiate between cognitive and mood symptoms, and is sensitive to treatment effects over a wide range of depression severity. The scale has nineteen items that are based on the week prior to the interview and which are rated as absent, mild or intermittent, and severe. Symptoms are clustered into five main categories: mood related signs, behavioural disturbance, physical signs, cyclic functions, and ideational disturbance. Published inter-rater reliability kappa is 0.67, internal consistency is reasonable (0.84), and it has been found to be valid, based on comparison with the Hamilton Depression Rating Scale and Research Diagnostic Criteria [38,39].

#### Quality of Life (QoL)

The quality of life will be measured with the *Qualidem*. The Qualidem is an easy to administer and sufficiently reliable and valid rating scale that provides a quality of life profile of persons with dementia in LTC settings. The Qualidem can be used for evaluation as well as for research and practice innovation. Twenty-one of 40 items are suitable for people with very severe dementia [40-42]. Each subscale of quality of life is scored. It is not possible to calculate a total score of the Qualidem. The individual item scores for each subscale are summed: the higher the score, the better the quality of life.

### Demographics, baseline and control variables

Sex, age and cultural background of residents will be derived as demographic variables from structured questionnaires filled in by nurses. A subscale of the Residents Assessment Inventory (RAI) will be used to obtain co-morbidity and will be filled in by the nursing home physicians. They will also determine the stage of dementia, measured with the Global Deterioration Scale [29,62] and the Cognitive Performance Scale (MDS-CPS).

The MDS-CPS is a seven-category index, ranging from cognitively intact to very severely impaired. The scale has shown excellent agreement with the Mini-Mental State Examination (MMSE) in the identification of cognitive impairment in research [63].

The Global Deterioration Scale (GDS) [29] consists of a seven-point scale (1-7) ranging from 'no global impairment' (1) to 'very severe global impairment' (7). Characteristics of the nursing homes (such as available disciplines, amount and nature of followed education by health care professionals, unit sizes) will be carefully registered and taken in to account when performing the analyses.

#### Medication

Changes in prescriptions of analgesics and psychotropic drugs (coded as defined daily dosage-DDD) will be derived from the daily logs of contact nurses and will also be derived from the analysis of pharmacists' electronic patient records for the total study period of 26 weeks.

#### Apolipoprotein Epsilon 4

The status of Apolipoprotein Epsilon 4 (Apo-E4) will be determined as a baseline variable and will be taken from two buccal mucosa swabs (Catch-All swabs of the firm BIOzymTC example). The Apo-E4 protein may play a role in the response to (pharmacological) treatments [64]. It is currently unclear whether the Apo-E4 protein has an effect on non-pharmacological treatments, such as comfort interventions. Determining the Apo-E4 is therefore very important in intervention studies like the present study.

### Process variables

The actual use of the STA OP!-protocol will be derived from the daily logs of contact nurses for the total study period of 26 weeks, and from group interviews with contact nurses and nursing home physicians in weeks 26. The researcher/research assistant making site-visits once a week, will check the thoroughness of the log completion by the contact nurse. After the research period the internal formed focus group will take over this task, in order to facilitate implementation.

#### Patient files

The use and nature of non-pharmacological comfort interventions, as well as the nature and number of physical restraints will be derived from daily logs by the contact nurses and by studying patient files for the total study period of 26 weeks.

### Statistical analyses

To assess effects of the intervention on outcome variables, we will perform multilevel analyses with statistical adjustments for differences in the relevant baseline scores and control variables according to the intention-to-treat principle. Where no confounding is to be expected, univariate analysis will be applied. Both an intention-to-treat analysis and per protocol analysis will be performed.

### Ethical approval

The study has been approved by the Medical Ethics Review Committee of the VU University Medical Center Amsterdam (registration number 2009/119).

## Discussion

This study will evaluate the use and effectiveness of the Dutch version of the STI-protocol. The transfer from the American design to the Dutch design has necessitated several changes due to of the different organisation of healthcare systems. Next to the similarities between the original STI and the Dutch version, e.g. the stepwise approach and essentially the same content of each step, there are some differences. For instance, the presence of a nursing home physician and psychologist adds clinical expertise to the team. It will be interesting to see if these differences will influence the effects of the intervention. This expertise is also available in the control setting. This might therefore lead to smaller differences between control and intervention then in the USA, but it might also prove to strengthen the stepwise approach, and show stronger effects. Furthermore, the question arises whether the addition of a standard observational pain scale contributes to pain assessment.

In our design are also some essential differences with the RCT Kovach undertook. We decided to use the CMAI and NPI-NH as behavioural outcome measures, and PACSLAC-D and MDS-RAI pain scale as pain measures. In the STI-study, the DS-DAT was used, which is essentially a discomfort scale. By adding measures for depression (MDS depression rating scale and Cornell) and quality of life (Qualidem) we believe we have the opportunity to study more precisely the effects of this stepwise protocol. To tackle the problem of using observational scales, which are best filled out by staff who know the patient well, yet who might be biased because of the intervention, we have added video recordings that will be scored by independent raters. We will also meticulously register the medication used, and interventions that are performed. By taking Buccal mucosa swabs, we will be able to study if there are responder differences caused by the Apo-E4 status.

A difficulty in research in nursing home care is that the usual care of the control groups is not standardized. Although nurses and nursing home physicians will have some routines and standard procedures in caring for residents with pain or affective discomfort, standard care will differ between nursing homes. Therefore, it is important that during the total assessment period all prescriptions of analgesics and psychotropic drugs, and non-pharmacological comfort interventions are carefully registered. Another difficulty in this study is the variability in the amount of training between the control and intervention condition. In the control condition the team receives a standardized training program focused on challenging behaviour and pain, which explicitly does not contain a stepwise approach. The intervention training is specifically designed to work with the stepwise STA OP!-method, and this stepwise approach results in extra training sessions. A confounder may therefore be the extra amount of attention in the intervention group, with a possible stronger Hawthorn effect.

In conclusion, pain and challenging behaviour are a major problem in moderate to severe dementia patients in nursing homes. This study aims to contribute to the evidence-based treatment of nursing home residents who experience discomfort, pain and behavioural symptoms.

## Competing interests

The authors declare that they have no competing interests.

## Authors' contributions

CK conceived and designed the original study (STI). AF and WA adjusted the STI study into STA OP! and obtained funding. MP drafted the manuscript and coordinates the data collection. AF, WA, ES and JS designed the STA OP!-study and helped to draft the manuscript. All authors have been involved in revising the manuscript. All authors read and approved the final manuscript.

## Pre-publication history

The pre-publication history for this paper can be accessed here:

http://www.biomedcentral.com/1471-2318/11/12/prepub

## References

[B1] Gezondheidsraad Dementie 2002 Den Haag publication number, 2002/004

[B2] de Lange J Poos MJJC Schoemaker C Hoe vaak komt dementie voor en hoeveel mensen sterven eraan? Volksgezondheid Toekomst Verkenning, Nationaal Kompas Volksgezondheid Bilthoven: RIVM http://www.nationaalkompas.nl/gezondheid-en-ziekte/ziekten-en-aandoeningen/psychische-stoornissen/dementie/omvang

[B3] Scherder EJ Bouma A Is decreased use of analgesics in Alzheimer disease due to a change in the affective component of pain? Alzheimer Disease and Related Disorders 1997 11 171 174 10.1097/00002093-199709000-00010 9305503

[B4] Sawyer P Lillis JP Bodner EV Allman RM Substantial daily pain among nursing home residents J Am Med Dir Assoc 2007 8 158 165 10.1016/j.jamda.2006.12.030 17349944

[B5] Boerlage AA van Dijk M Stronks DL de Wit R van der Rijt CC Pain prevalence and characteristics in three Dutch residential homes European Journal of Pain 2008 12 7 910 6 10.1016/j.ejpain.2007.12.014 18267371

[B6] Achterberg WP Gambassi G Finne-Soveri H Liperoti R Noro A Frijters DH Cherubini A Dell'aquila G Ribbe MW Pain in European long-term care facilities: Cross-national study in Finland, Italy and the Netherlands Pain 2010 148 70 74 10.1016/j.pain.2009.10.008 19910119

[B7] American Geriatrics Society Panel on Chronic Pain in Older Persons The management of chronic pain in older persons: American Geriatrics Society Panel on Chronic Pain in Older Persons J Am Geriatr Soc 1998 46 635 651 9588381 10.1111/j.1532-5415.1998.tb01084.x

[B8] Kovach CR Noonan PE Griffie J Muchka S Weissman DE Use of the ADD protocol Appl Nurs Res 2001 4 4 193 200 10.1053/apnr.2001.26784 11699022

[B9] Zuidema SU de Jonghe JF Verhey FR Koopmans RT Neuropsychiatric symptoms in nursing home patients: factor structure invariance of the Dutch nursing home version of the neuropsychiatric inventory in different stages of dementia Dement Geriatr Cogn Disord 2007 24 3 169 76 10.1159/000105603 17641527

[B10] Kverno K Rabins P Blass D Hicks K Black B Prevalence and treatment of neuropsychiatric symptoms in hospice-eligible nursing home residents with advanced dementia Journal of Gerontological Nursing 34 12 8 15 10.3928/00989134-20081201-03 19112999 PMC2828146

[B11] Norton MJ Allen RS Snow AL Hardin JM Burgio LD Predictors of need-driven behaviors in nursing home residents with dementia and associated certified nursing assistant burden Aging and Mental Health 2010 14 3 303 309 10.1080/13607860903167879 20425649

[B12] Frampton M Experience assessment and management of pain in people with dementia Age Ageing 2003 32 248 51 10.1093/ageing/32.3.248 12720608

[B13] Nygaard HA Jarland M Are nursing home patients with dementia diagnosis at increased risk for inadequate pain treatment? International Journal of Geriatric Psychiatry 2005 20 5 730 737 10.1002/gps.1350 16035124

[B14] Achterberg W Pot AM Scherder E Ribbe M Pain in the nursing home: assessment and treatment on different types of care wards J Pain Symptom Manage 2007 34 5 480 487 10.1016/j.jpainsymman.2006.12.017 17616332

[B15] Tait RC Chibnall JT Under-treatment of pain in dementia: assessment is key J Am Med Dir Assoc 2008 9 6 372 4 10.1016/j.jamda.2008.04.001 18585637

[B16] Feldt KS Warden MA Ryden MB Examining pain in aggressive cognitively impaired older adults J of Gerontological Nursing 1998 24 11 14 22 10.3928/0098-9134-19981101-07 10392090

[B17] Herr K Chronic pain in the older patient: management strategies J Gerontol Nurs 2002 28 2 28 34 11846288 10.3928/0098-9134-20020201-08

[B18] Horgas Pain Management in Elderly Adults Journal of Infusion Nursing 2003 26 3 161 165 2003 10.1097/00129804-200305000-00007 12792374

[B19] Geda YE Rummans TR Pain: cause of agitation in elderly individuals with dementia Am J Psychiatry 1999 156 10 1662 3 10518184 10.1176/ajp.156.10.1662-a

[B20] Briesacher BA Limcangco MR Simoni-Wastila L Doshi JA Levens SR Shea DG Stuart B The quality of antipsychotic drug prescribing in nursing homes Arch Intern Med 2005 165 11 1280 5 10.1001/archinte.165.11.1280 15956008

[B21] Kovach CR Weissman DE Griffie J Matson S Muchka S Assessment and treatment of discomfort for people with late-stage dementia J Pain Symptom Manage 1999 18 6 412 419 10.1016/S0885-3924(99)00094-9 10641467

[B22] Kovach CR Noonan PE Griffie J Muchka S Weissman DE The assessment of discomfort in dementia protocol Pain Manag Nurs 2002 3 1 16 27 10.1053/jpmn.2002.30389 11893998

[B23] Kovach CR Logan BR Noonan PE Schlidt AM Smerz J Simpson M Wells T Effects of the serial trial intervention on discomfort and behavior of nursing home residents with dementia Am J Alzheimers Dis Other Demen 2006 21 3 147 55 10.1177/1533317506288949 16869334 PMC10833286

[B24] Algase DL Beck C Kolanowski A Whall A Berent S Richards K Beattie E Needdriven dementia-compromised behavior: An alternative view of disruptive behavior American Journal of Alzheimer's Disease 1996 11 10 19

[B25] Kovach CR Noonan PE Schlidt AM Wells T A model of consequences of need-driven, dementia-compromised behavior J Nurs Scholarsh 2005 37 2 134 40 discussion 140. Review 10.1111/j.1547-5069.2005.00025_1.x 15960057

[B26] Hurley AC Volicer BJ Hanrahan PA Houde S Volicer L Assessment of discomfort in advanced Alzheimer patients Res Nurs Health 1992 15 5 369 77 10.1002/nur.4770150506 1529121

[B27] Ribbe MW Ljunggren G Steel K Topinková E Hawes C Ikegami N Henrard JC Jónnson PV Nursing homes in 10 nations: a comparison between countries and settings Age Ageing 1997 26 Suppl 2 3 12 9464548 10.1093/ageing/26.suppl_2.3

[B28] Conroy S Van Der Cammen T Schols J Van Balen R Peteroff P Luxton T Medical services for older people in nursing homes--comparing services in England and the Netherlands J Nutr Health Aging 2009 13 6 559 63 10.1007/s12603-009-0107-9 19536425 PMC12880319

[B29] Reisberg B Ferris S de Leon MJ Crook T The Global Deterioration Scale (GDS) for assessment of primary degenerative dementia Am J Psychiatry 1982 139 1136 1139 7114305 10.1176/ajp.139.9.1136

[B30] Cohen-Mansfield J Marx MS Rosenthal AS A description of agitation in the nursing home J Gerontol 1989 44 77 84 10.1093/geronj/44.3.m77 2715584

[B31] de Jonghe JFM Kat MG Factor structure and validity of the Dutch version of the Cohen-Mansfield Agitation Inventory (CMAI-D) Journal of the American Geriatrics Society 1996 44 7 888 889 8675952 10.1111/j.1532-5415.1996.tb03762.x

[B32] Cummings JL Mega M Gray K Rosenberg-Thompson S Carusi DA Gornbein J The Neuropsychiatric Inventory: comprehensive assessment of psychopathology in dementia Neurology 1994 44 12 2308 14 7991117 10.1212/wnl.44.12.2308

[B33] Cummings JL The Neuropsychiatric Inventory: assessing psychopathology in dementia patients Neurology 1997 48 suppl 6 S10 S16 9153155 10.1212/wnl.48.5_suppl_6.10s

[B34] Fries BE Simon SE Morris JN Flodstrom C Bookstein FL Pain in U.S. Nursing Homes: Validating a Pain Scale for the Minimum Data Set The Gerontologist 2001 41 2 173 179 11327482 10.1093/geront/41.2.173

[B35] Fuchs-Lacelle S Hadjistavropoulos T Development and preliminary validation of the pain assessment checklist for seniors with limited ability to communicate (PACSLAC) Pain Manag Nurs 2004 5 37 49 10.1016/j.pmn.2003.10.001 14999652

[B36] Zwakhalen SM Hamers JP Abu-Saad HH Berger MP Pain in elderly people with severe dementia: a systematic review of behavioural pain assessment tools BMC Geriatr 2006 6 3 10.1186/1471-2318-6-3 16441889 PMC1397844

[B37] Zwakhalen SMG Hamers JPH Berger MPF Improving the clinical usefulness of a behavioural pain scale for older people with dementia Journal of Advanced Nursing 2007 58 5 493 502 10.1111/j.1365-2648.2007.04255.x 17442027

[B38] Conn D Thorpe L Assessment of behavioural and psychological symptoms associated with dementia Can J Neurol Sci 2007 34 Suppl 1 S67 71 17469686 10.1017/s0317167100005606

[B39] Dröes RM Cornell Scale for Depression in Dementia 1993 Nederlandse vertaling. Vakgroep Psychiatrie, Vrije Universiteit, Amsterdam

[B40] Ettema TP Dröes RM de Lange J Mellenbergh GJ Ribbe MW QUALIDEM: development and evaluation of a dementia specific quality of life instrument. Scalability, reliability and internal structure Int J Geriatr Psychiatry 2007 22 6 549 56 10.1002/gps.1713 17152121

[B41] Ettema TP Dröes RM de Lange J Mellenbergh GJ Ribbe MW QUALIDEM: development and evaluation of a dementia specific quality of life instrument--validation Int J Geriatr Psychiatry 2007 22 5 424 30 10.1002/gps.1692 17044131

[B42] Schölzel-Dorenbos CJ Ettema TP Bos J Boelens-van der Knoop E Gerritsen DL Hoogeveen F de Lange J Meihuizen L Dröes RM Evaluating the outcome of interventions on quality of life in dementia: selection of the appropriate scale Int J Geriatr Psychiatry 2007 22 6 511 9 17133655 10.1002/gps.1719

[B43] Katz S Down TD Cash HR Progress in the Development of the Index of ADL Gerontologist 1970 10 20 30 5420677 10.1093/geront/10.1_part_1.20

[B44] Zuidema SU Derksen E Verhey FR Koopmans RT Prevalence of neuropsychiatric symptoms in a large sample of Dutch nursing home patients with dementia Int J Geriatr Psychiatry 2007 22 7 632 8 10.1002/gps.1722 17136713

[B45] Eldridge SM Ashby D Kerry S Sample size for cluster randomized trials: effect of coefficient of variation of cluster size and analysis method Int J epidem 2006 35 1292 1300 10.1093/ije/dyl129 16943232

[B46] van Weert JC van Dulmen AM Spreeuwenberg PM Ribbe MW Bensing JM Behavioral and mood effects of snoezelen integrated into 24-hour dementia care J Am Geriatr Soc 2005 53 1 24 33 10.1111/j.1532-5415.2005.53006.x 15667372

[B47] World Health Organization Pain Ladder http://www.who.int/cancer/palliative/painladder

[B48] Ypma-Bakker MEM Glas ER Hagens JHAM NVVA-richtlijn Probleemgedrag. Tijdschrift Verpleeghuisgeneeskunde 2002 26 5 31 revised december 2008: Smalbrugge M etal. 2008

[B49] Finkel S Lyons JS Anderson RL Reliability and validity of the Cohen-Mansfield Agitation Inventory in institutionalized elderly Int J Geriatr Psychiatry 1992 7 487 90 10.1002/gps.930070706

[B50] Koss E Weiner M Ernesto C Cohen-Mansfield J Ferris SH Grundman M Schafer K Sano M Thal LJ Thomas R Whitehouse PJ Assessing patterns of agitation in Alzheimer's disease patients with the Cohen-Mansfield Agitation Inventory The Alzheimer's Disease Cooperative Study. Alzheimer Dis Assoc Disord 1997 11 Suppl 2 S45 50 10.1097/00002093-199700112-00007 9236952

[B51] Wood S Cummings JL Hsu MA Barclay T Wheatley MV Yarema KT Schnelle JF The use of the Neuropsychiatric Inventory in nursing home residents: Characterization and measurement American Journal of Geriatric Psychiatry 2000 8 75 83 10.1097/00019442-200002000-00010 10648298

[B52] Kat MG de Jonghe JFM Aalten P Kalisvaart CJ Droes RM Verhey FRJ Neuropsychiatric symptoms of dementia: psychometric aspects of the Dutch Neuropsychiatric Inventory (NPI) Tijdschr Gerontol Geriatr 2002 33 150 155 12378786

[B53] Koopmans RT van der Molen M Raats M Ettema TP Neuropsychiatric symptoms and quality of life in patients in the final phase of dementia Int J Geriatr Psychiatry 2009 24 1 25 32 10.1002/gps.2040 18512257

[B54] Morris JN Murphy K Nonemaker S Long term care facility Resident AssessmentInstrument (RAI) user's manual version 2.0 1995 Baltimore: HCFA

[B55] van der Steen JT Ooms ME van der Wal G Ribbe MW Het meten van (on)welbevinden bij demente patiënten Tijdschr Gerontol Geriatr 2002 33 6 257 63 12611289

[B56] Hoogendoorn LI Kamp S Mahomed CA Adèr HJ Ooms ME van der Steen JT De rol van de observator in de betrouwbaarheid van de Nederlandse versie van de Discomfort Scale-Dementia of Alzheimer Type (DS-DAT) Tijdschr Gerontol Geriatr 2001 32 3 117 21 11455871

[B57] Van der Steen JT Adèr HJ van Assendelft JH Kooistra M Passier PE Ooms ME Retrospectieve afname van de Nederlandse versie van de Discomfort Scale--Dementia of Alzheimer type (DS-DAT): is inschatten achteraf voldoende valide en betrouwbaar? Tijdschr Gerontol Geriatr 2003 34 6 254 9 15007957

[B58] Kunz M Scharmann S Hemmeter U Schepelmann K Lautenbacher S The facial expression of pain in patients with dementia Pain 2007 133 1-3 221 8 10.1016/j.pain.2007.09.007 17949906

[B59] Burrows AB Morris JN Simon SE Hirdes JP Phillips C Development of a minimum data set-based depression rating scale for use in nursing homes Age Ageing 2000 29 2 165 72 10.1093/ageing/29.2.165 10791452

[B60] Morris JN Hawes C Fries BE Designing the National Resident Assessment Instrument for Nursing Homes The Gerontologist 1990 30 293 307 2354790 10.1093/geront/30.3.293

[B61] Morris JN Nonemaker S Murphy K Hawes C Fries BE Mor V Phillips C A commitment to change: revision of HCFA's RAI J Am Geriatr Soc 1997 45 8 1011 6 9256856 10.1111/j.1532-5415.1997.tb02974.x

[B62] Reisberg B Ferris SH Franssen E An ordinal functional assessment tool for Alzheimer's-type dementia Hospital and Community Psychiatry 1985 36 593 595 4007814 10.1176/ps.36.6.593

[B63] Hartmaier SL Sloane PD Guess HA Koch GG Mitchell CM Phillips CD Validation of the Minimum Data Set Cognitive Performance scale: agreement with the mini-mental state examination J Gerontol Med Sci 1995 50 M128 33 10.1093/gerona/50a.2.m128 7874589

[B64] Salloway S Sperling R Gilman S Fox NC Blennow K Raskind M Sabbagh M Honig LS Doody R van Dyck CH Mulnard R Barakos J Gregg KM Liu E Lieberburg I Schenk D Black R Grundman M Bapineuzumab 201 Clinical Trial Investigators. A phase 2 multiple ascending dose trial of bapineuzumab in mild to moderate Alzheimer disease Neurology 2009 73 24 2061 70 10.1212/WNL.0b013e3181c67808 19923550 PMC2790221

